# Starvation-induced HSC70 O-GlcNAcylation activates chaperone-mediated autophagy

**DOI:** 10.1016/j.jbc.2026.113165

**Published:** 2026-05-15

**Authors:** Ningda Xu, Xiangpeng Wang, Jiyue Zhang, Jianxin Zhao, Sheng Yan, Wen Zhou, Wei Chi, Jing Li

**Affiliations:** 1 Shenzhen Eye Hospital, Shenzhen Eye Medical Center, Southern Medical University, Shenzhen, Guangdong, China; 2 Beijing Key Laboratory of DNA Damage Response and College of Life Sciences, Capital Normal University, Beijing, China; 3 College of Chemistry and Molecular Engineering, Peking University, Beijing, China

**Keywords:** Ataxin-10, chaperone-mediated autophagy, HSC70, O-GlcNAc, starvation

## Abstract

O-linked β-N-acetylglucosamine (O-GlcNAc) functions as a nutrition rheostat to mediate cellular signaling pathways. It fluctuates in response to various nutritional factors, for instance, glucose availability. Previous investigations have shown that glucose deprivation upregulates O-GlcNAcylation levels. Meanwhile, starvation also activates autophagy, in particular, chaperone-mediated autophagy (CMA). But it is unknown what signal activates CMA during starvation. In the CMA pathway, heat shock cognate 70 kDa protein (HSC70) recognizes client proteins that bear a KFERQ pentapeptide motif, and delivers them for lysosomal degradation. Herein, we show that glucose depletion increases both the affinity between HSC70 and O-GlcNAc transferase, and HSC70 O-GlcNAcylation levels. We validated that HSC70 is O-GlcNAcylated at T430 according to a previous chemoproteomic screen. We further demonstrate that O-GlcNAcylation attenuates HSC70 stability, but increases its binding with known CMA substrates, such as PKM2. We thus posit that starvation-induced HSC70 O-GlcNAcylation may activate CMA. To test this, we used label-free quantitative mass spectrometry to analyze HSC70-WT and HSC70-T430A interactome, and obtained a proteome-wide potential CMA substrate pool. By studying this dataset, we identified a new CMA substrate, Ataxin-10, a protein involved in a neurologic disorder. We then validated our model by mapping a potential KFERQ motif on Ataxin-10 and showing that HSC70-T430A decreased binding with Ataxin-10. In sum, our work suggests that CMA and O-GlcNAcylation intersect at HSC70, and starvation-induced O-GlcNAcylation of HSC70 is part of the signal that activates CMA during fasting.

O-linked β-N-acetylglucosamine (O-GlcNAc) modification occurs on the Ser or Thr residues of proteins and functions as a rheostat in response to nutrient status (1). It is catalyzed by O-GlcNAc transferase (OGT) and removed by O-GlcNAcase (OGA) in humans. It is well recognized that the dynamic and reversible O-GlcNAcylation contributes to signal transduction in many biological processes in both health and disease (2, 3). Vice versa, O-GlcNAcylation levels would rise and fall in response to cellular nutrient status. Low glucose or fasting, for instance, would upregulate O-GlcNAcylation levels in various types of human cells and the fly, probably due to elevated OGT mRNA levels (4, 5, 6, 7). Equally important, the glucose-sensitive Unc-51 like autophagy activating kinase 1 (ULK1) phosphorylates OGT on glucose depletion and upregulates OGT protein levels (8). These studies shed light on the regulation of OGT upon stress signals, in particular, when glucose is lacking.

Depleted glucose also activates autophagy. Autophagy refers to the process of lysosomal degradation of cytoplasmic components, and the nutrients or energy thus generated would allow cell survival in times of need (9). Autophagy dysfunction is linked to abnormalities in differentiation and development, and many human diseases including neurodegenerative disorders (10, 11, 12). Broadly categorized, autophagy encompasses macroautophagy, microautophagy, and chaperone-mediated autophagy (CMA) (10). Client proteins of CMA bear a KFEQR motif, and are thus recognized by heat shock cognate protein 70 (HSC70) and delivered to lysosome-associated membrane protein type 2A (Lamp2a) on the lysosome. Subsequently, substrate proteins are degraded by the lysosome (13, 14).

As HSC70 is pivotal for CMA selectivity and specificity, it is not surprising that it is regulated at multiple levels (15). The vast array of posttranslational modifications (PTMs), such as acetylation, phosphorylation, and ubiquitination, supports a chaperone code hypothesis, in which PTMs fine-tune HSC70 chaperone activity, localization, and stability (15). HSC70 acetylation, for instance, enhances CMA activity and alleviates cellular senescence (16), and acetylation has been widely recognized as a vital factor regulating autophagy (17). Hsc70 is also phosphorylated at Ser, Thr, or Tyr (18, 19), among many other PTMs.

In sharp contrast, little is known about the role of O-GlcNAc in regulating CMA (15). Previously, the autophagy initiating kinase ULK1 is shown to be degraded *via* CMA (20), and O-GlcNAcylation stabilizes ULK1 by inhibiting CMA upon human papillomavirus invasion (21). In addition, OGA is subject to SUMOylation, and subsequently targeted to CMA (22, 23). But how OGT intertwines with HSC70 or the CMA pathway remains a mystery. In parallel, CMA is activated upon starvation, but what is the signal that activates CMA during fasting?

Herein, we demonstrate that HSC70 binds with OGT, and the interaction increases upon glucose depletion when CMA is activated. HSC70 is O-GlcNAcylation at T430, a residue in its hydrophobic pocket. HSC70 O-GlcNAcylation not only downregulates its stability, but also alters its interactome, as label-free quantitative mass spectrometry (MS) revealed that wild-type HSC70 interacts with many proteins at a higher abundance than HSC70-T430A. Among the interacting proteins, we further found a new CMA substrate, Ataxin-10, and identified its potential KFERQ motif. Collectively, our work deepened the link between O-GlcNAc and CMA, and suggests that HSC70 O-GlcNAcylation regulates its stability and CMA activity according to sugar levels.

## Results

### Glucose deprivation elevates the interaction between HSC70 and OGT

**Figure 1:**
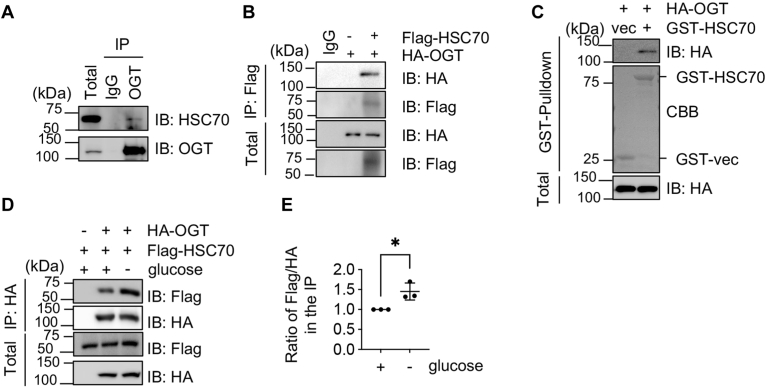
**Glucose deprivation elevates the interaction between OGT and HSC70.***A*, co-immunoprecipitation between endogenous OGT and HSC70. *B*, HEK-293T cells were transfected with HA-OGT and Flag-HSC70 plasmids, and then cellular lysates were immunoprecipitated with anti-Flag antibodies and immunoblotted with anti-HA and anti-Flag antibodies. *C*, cells were transfected with HA-OGT plasmids. Recombinant GST-HSC70 proteins were incubated with the cellular lysates, and GST pull-down experiments were carried out. *D*, cells were transfected with HA-OGT and Flag-HSC70 plasmids, treated with glucose or not, and then the lysates were immunoprecipitated with anti-HA antibodies and immunoblotted with antibodies indicated. *E*, the quantitation of (*D*). The statistical analysis in (*E*) was performed using Student’s *t* test. ∗*p* < 0.05. OGT, O-GlcNAc transferase; HSC70, heat shock cognate protein 70.

We are interested in identifying potential PTMs on HSC70. A previous proteomic screen demonstrated that the O-GlcNAcylation of HSC70 occurs at T430 (24), so it prompted us to examine whether HSC70 is O-GlcNAcylated. We first assessed the potential biochemical interaction between OGT and HSC70. Cell lysates were immunoprecipitated with anti-OGT antibodies, and HSC70 was detected in the immunoprecipitates (Fig. 1*A*). We also examined the interaction between overproduced proteins (Fig. 1*B*). Cells were transfected with Flag-HSC70 and HA-OGT plasmids, then the lysates were immunoprecipitated with anti-Flag antibodies. The results showed that HA-OGT co-immunoprecipitations with Flag-HSC70. Recombinant GST-HSC70 proteins were also utilized in pull-down assays (Fig. 1*C*). Cells were transfected with HA-OGT, and the cellular extracts were incubated with recombinant GST-HSC70 proteins (Fig. 1*C*). GST-HSC70 could pull-down HA-OGT (Fig. 1*C*). These results suggest that HSC70 and OGT associate with each other.

Previous investigations demonstrated that cellular O-GlcNAcylation levels increase upon glucose deprivation (4, 5, 6, 8). As autophagy is also activated when nutrient is scarce, we then detected the interaction between HSC70 and OGT under glucose deprivation conditions, and observed that the interaction between OGT and HSC70 elevated significantly when glucose is absent (Fig. 1, *D* and *E*). These results suggest that glucose deprivation promotes the interaction between OGT and HSC70.

### Glucose depletion increases HSC70 O-GlcNAcylated at T430

**Figure 2:**
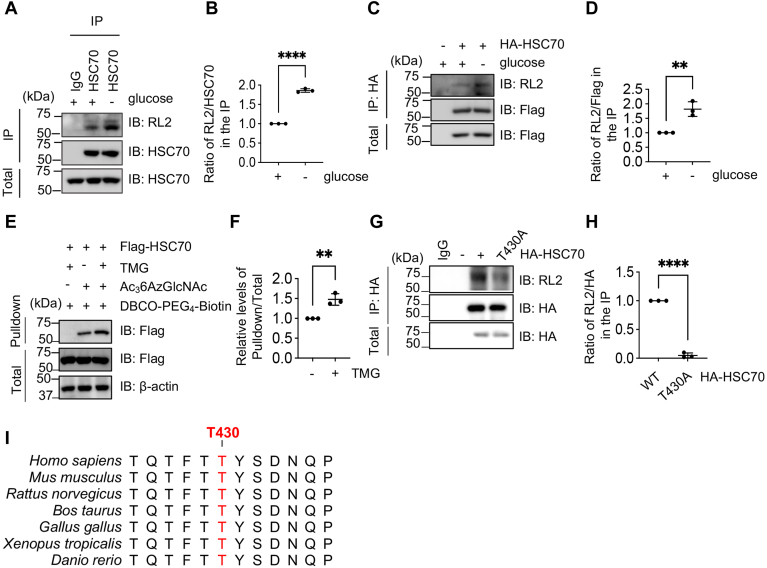
**Glucose depletion enhances HSC70 O-GlcNAcylated at T430.***A*, the O-GlcNAcylation modification of endogenous HSC70 was detected under conditions of glucose deprivation or no treatment. *B*, the quantitation of (*A*). *C*, cells were transfected with HA-OGT and its O-GlcNAcylation was detected under conditions of glucose deprivation or no treatment. *D*, the quantitation of (*C*). *E*, cells were transfected with Flag-HSC70, then treated with 200 μmol/L Ac_3_6AzGlcNAc or not treated, and treated with 5 μmol/l TMG or not, as previously described (25). *F*, the quantitation of (*E*). *G*, HEK-293T cells were transfected with HA-HSC70-WT and HA-HSC70-T430A, and then the lysates were immunoprecipitated and immunoblotted with the antibodies indicated. *H*, the quantitation of (*G*). *I*, T430 of HSC70 is conserved in multiple species. The statistical analysis was performed using Student’s *t* test. ∗∗*p* < 0.01; ∗∗∗∗*p* < 0.0001. HSC70, heat shock cognate protein 70; O-GlcNAc, O-linked β-N-acetylglucosamine; OGT, O-GlcNAc transferase; TMG, Thiamet-G.

We sought to test HSC70 O-GlcNAcylation, especially under glucose depleted conditions. When the cells were glucose deprived for 24 h, it could be observed that endogenous HSC70 O-GlcNAcylation increased robustly (Fig. 2, *A* and *B*). We also observed that O-GlcNAcylation of overexpressed HA-HSC70 increases when glucose is deprived (Fig. 2, *C* and *D*). We then utilized click chemistry as previously described (25). Cells were transfected with HA-HSC70 plasmids and incubated with Ac_3_6AzGlcNAc. The lysates were subsequently incubated with DBCO-PEG_4_-Biotin. As shown in Figure 2, *E* and *F*, the pull-down experiments further demonstrated that HSC70 is O-GlcNAcylated.

As a previous proteomic screen identifies HSC70 O-GlcNAcylation at T430 (24), we generated HSC70-T430A plasmids accordingly. HA-HSC70-WT shows RL2 staining in the IB, but HA-HSC70-T430A mutants completely abolish RL2 signals (Fig. 2, *G* and *H*). Sequence alignment shows that HSC70 T430 is conserved in various organisms (Fig. 2*I*). Another proteomic screen suggests that HSC70 is O-GlcNAcylated at T502 (26), but our mutagenesis study indicates that it has little effects on HSC70 O-GlcNAcylation (Fig. S1). Taken together, our results suggest that HSC70 O-GlcNAcylated elevates upon glucose deprivation with a major modification site to be T430.

### HSC70 O-GlcNAcylation promotes ubiquitination

**Figure 3:**
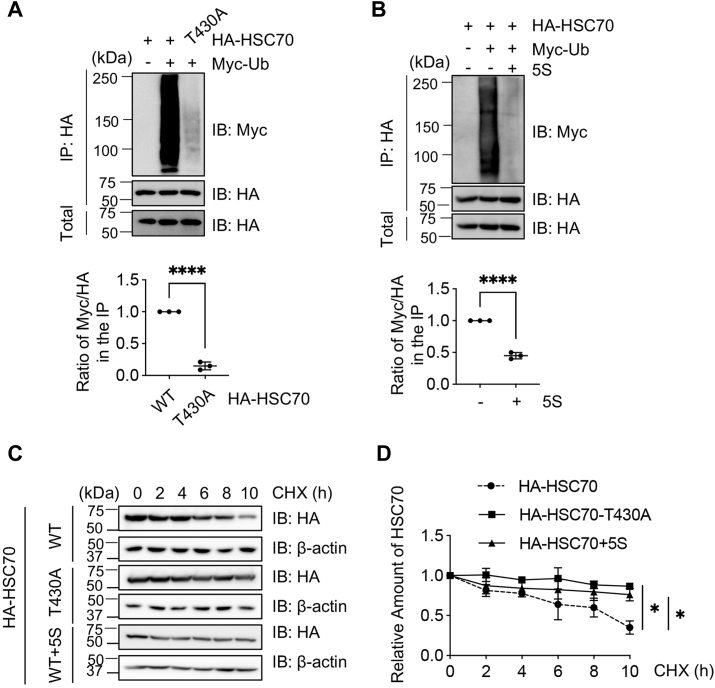
**HSC70 O-GlcNAcylation promotes ubiquitination.***A*, HEK-293T cells were transfected with HA-HSC70-WT, -T430A, and Myc-Ub plasmids, and then the lysates were immunoprecipitated and immunoblotted with the antibodies indicated. The ratio of Myc/HA in the IP was quantified and shown in the bottom panel. *B*, cells were transfected with HA-HSC70-WT and Myc-Ub, then treated with 50 μmol/L acetyl-5S-GlcNAc (5S, OGT inhibitor) or not treated. The ratio of Myc/HA in the IP was quantified and shown in the bottom panel. *C* and *D*, cycloheximide (CHX) pulse-chase assays. Cells were transfected with HA-HSC70-WT, HA-HSC70-T430A plasmids, treated or untreated with 5S, then treated with CHX for different durations. The quantitation is in (*D*). A two-way ANOVA test was used for statistical analysis. ∗*p* < 0.05. The statistical analysis in (*A*) and (*B*) was performed using Student’s *t* test. ∗∗∗∗*p* < 0.0001. HSC70, heat shock cognate protein 70; IP, immunoprecipitation; O-GlcNAc, O-linked β-N-acetylglucosamine; OGT, O-GlcNAc transferase.

It has been reported that HSC70 is degraded through the ubiquitin-proteasome pathway (15) and O-GlcNAcylation often regulates protein stability (27). Therefore, we examined HSC70 ubiquitination levels. As shown in Figure 3*A*, cells were transfected with HA-HSC70-WT, -T430A, and Myc-Ub plasmids, and the ubiquitination levels of HSC70 were assayed. The results showed that the HSC70-T430A mutant greatly diminished ubiquitination. To preclude the possibility that it is caused by the mutated residue, we then measured HSC70 ubiquitination using the chemical OGT inhibitor acetyl-5S-GlcNAc (5S) (Fig. 3*B*). Upon OGT inhibition, HSC70 attenuated ubiquitination markedly, suggesting that it is indeed the O-GlcNAcylation at T430 that modulates HSC70 ubiquitination. Moreover, we assessed endogenous Ub levels of HSC70 (Fig. S2), and found that endogenous Ub results are consistent with overproduced Myc-Ub levels. Furthermore, RL2 staining revealed that O-GlcNAcylation levels decreased simultaneously with the Ub levels (Fig. S2). These results indicate that HSC70 O-GlcNAcylation promotes ubiquitination.

Then, we employed cycloheximide (CHX) pulse-chase experiments. Cells were transfected with HA-HSC70-WT and HA-HSC70-T430A plasmids or HA-HSC70-WT plasmids plus 5S. Then cells were treated with CHX to inhibit new protein synthesis. The cellular lysates were collected at different time points to examine protein stability. As shown in Figure 3, *C* and *D*, the O-GlcNAc-deficient T430A mutant and WT+5S increased protein half-life compared to WT, consistent with the ubiquitination assay results. In sum, our biochemical results show that O-GlcNAcylation destabilizes HSC70, probably through enhanced ubiquitination.

### HSC70 O-GlcNAcylation promotes interaction with PKM2, one CMA client protein

It is known that HSC70 interacts with CMA client proteins and targets them to lysosomal degradation. T420 sits at the core of HSC70, hinting that O-GlcNAcylation may mediate the affinity between HSC70 and its clients. Therefore, we first evaluated the interaction between HSC70 mutants with reported CMA clients, such as pyruvate kinase M2 (PKM2).

**Figure 4:**
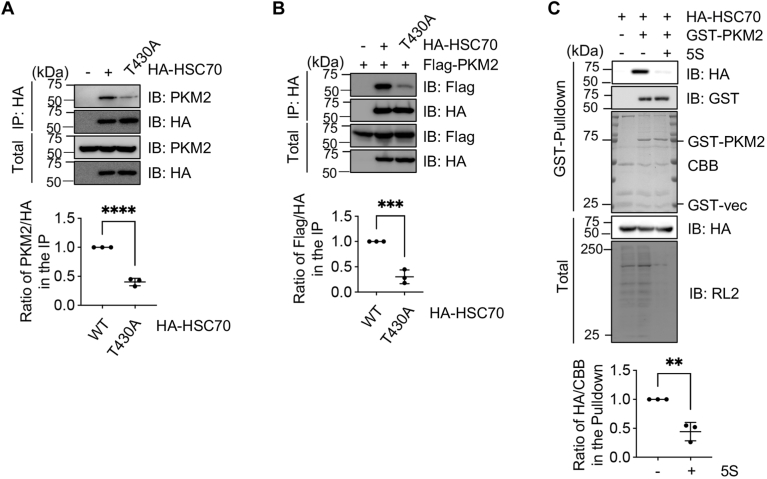
**HSC70 O-GlcNAcylation promotes interaction with PKM2, a CMA client protein.***A*, cells were transfected with HA-HSC70-WT and HA-HSC70-T430A plasmids, and then the lysates were immunoprecipitated and immunoblotted with the antibodies indicated. The ratio of PKM2/HA in the IP was quantified and shown in the bottom panel. *B*, cells were transfected with HA-HSC70-WT, -T430A and Flag-PKM2, and then the lysates were immunoprecipitated and immunoblotted with the antibodies indicated. The ratio of Flag/HA in the IP was quantified and shown in the bottom panel. *C*, cells were transfected with HA-HSC70, then treated with 5S or not treated. Recombinant GST-PKM2 proteins were incubated with the cellular lysates, and GST pull-down experiments were carried out. CBB stands for Coomassie brilliant blue. The ratio of HA/CBB in the pull-down was quantified and shown in the *bottom* panel. The statistical analysis was performed using Student’s *t* test. ∗∗*p* < 0.01; ∗∗∗*p* < 0.001; ∗∗∗∗*p* < 0.0001. CMA, chaperone-mediated autophagy; HSC70, heat shock cognate protein 70; IP, immunoprecipitation; O-GlcNAc, O-linked β-N-acetylglucosamine; PKM2, pyruvate kinase M2.

PKM2 has been reported to be degraded *via* the CMA pathway (28). We tested the interaction between HSC70 and PKM2 and found that HA-HSC70-T430A bound less endogenous PKM2 and Flag-PKM2 in cells (Fig. 4, *A* and *B*). We also used pull-down assays (Fig. 4*C*). Cells were transfected with HA-HSC70 and treated with 5S or not. The cellular lysates were incubated with recombinant GST-PKM2 proteins. After the 5S treatment, the amount of HA-HSC70 being pulled down decreased discernably (Fig. 4*C*). These results indicate that the O-GlcNAcylation modification at T430 of HSC70 underlies CMA client recognition.

### Label-free quantitative mass spectrometry identifies Ataxin-10 as a new CMA client protein

We wondered whether the interaction between HSC70-T430 O-GlcNAcylation modification and CMA client proteins hold true to other substrates, so we performed a label-free quantitative MS analysis of the HA-HSC70-WT and HA-HSC70-T430A interactome (Table S1). Among the proteins identified in the MS results is Ataxin-10. Ataxin-10 is encoded by the *ATXN10* gene, and ATTCT pentanucleotide-repeat expansion (up to 22.5 kb) in the gene is linked to spinocerebellar ataxia type 10 (SCA10), an autosomal dominant neurologic disorder (29, 30). Previously, we have shown that Ataxin-10 is phosphorylated by polo-like kinase 1 (PLK1) (31) and Aurora B (32) to regulate cytokinesis. Ataxin-10 is also shown to be localized in the Golgi (33), but it has not been reported to undergo degradation *via* the CMA pathway.

**Figure 5:**
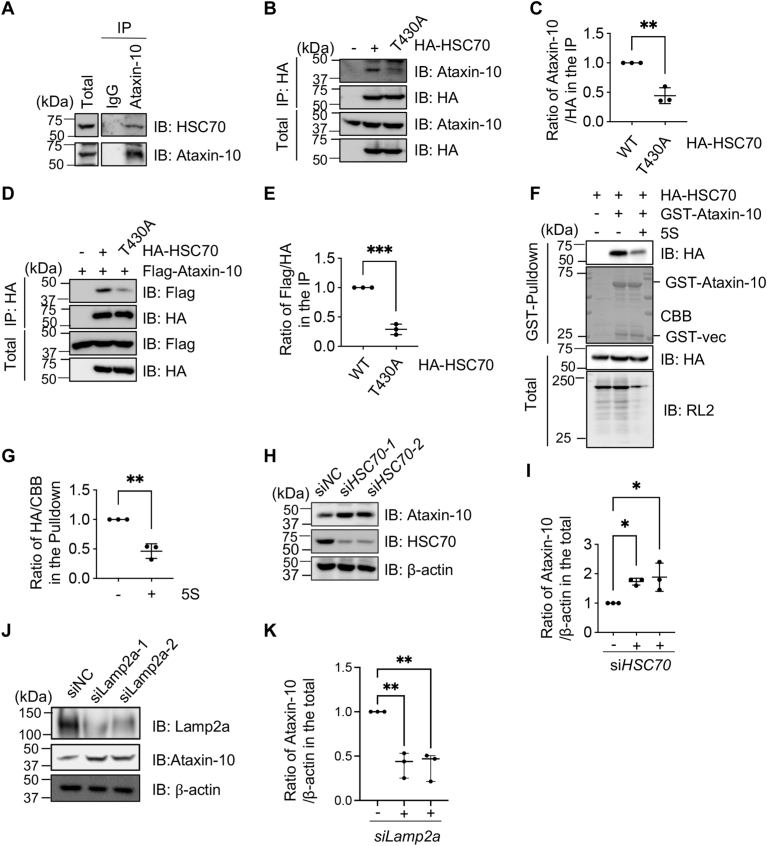
**Ataxin-10 is a CMA client protein.***A*, co-IP between endogenous Ataxin-10 and HSC70. *B*, cells were transfected with HA-HSC70-WT and HA-HSC70-T430A, and then the lysates were immunoprecipitated and immunoblotted with the antibodies indicated. The ratio of Ataxin-10/HA in the IP was quantified and shown in (*C*). *D*, cells were transfected with HA-HSC70-WT, -T430A, and Flag-Ataxin-10, and then the lysates were immunoprecipitated and immunoblotted with the antibodies indicated. The ratio of Flag/HA in the IP was quantified and shown in the bottom panel. *E*, quantitation of (*D*). *F*. cells were transfected with HA-HSC70, then treated with 5S or not treated. Recombinant GST-Ataxin-10 proteins were incubated with the cellular lysates, and GST pull-down experiments were carried out. The ratio of HA/CBB in the pull-down was quantified and showed in (*G*). *H*, cells were transfected with 2 siRNAs targeting *HSC70*. The quantitation was shown at (*I*). *J*, cells were transfected with 2 siRNAs targeting *Lamp2a*, and then the lysates were immunoblotted with the antibodies indicated. *K*, quantitation of the results in (*J*). The statistical analysis was performed using Student’s *t* test. ∗*p* < 0.05; ∗∗*p* < 0.01; ∗∗∗*p* < 0.001. CMA, chaperone-mediated autophagy; co-IP, co-immunoprecipitation; HSC70, heat shock cognate protein 70; IP, immunoprecipitation; Lamp2a, lysosome-associated membrane protein type 2A.

We first assessed the interaction between Ataxin-10 and HSC70. Endogenous Ataxin-10 and HSC70 were detected to form a complex (Fig. 5*A*). We examined the interaction between Ataxin-10 and overproduced HA-HSC70. Not only does Ataxin-10 interact with overproduced HSC70, but also HSC70-T430A decreases interaction with both endogenous and overproduced Ataxin-10 (Fig. 5, *B*–*E*). We also evaluated the interaction between HA-HSC70 and GST-Ataxin-10 after the cells were treated with the 5S inhibitor. After the treatment, the amount of HA-HSC70 being pulled down decreased significantly (Fig. 5, *F* and *G*). Next, we knocked down HSC70 with two independent si*HSC70* oligos, and Ataxin-10 accumulated in these cells (Fig. 5, *H* and *I*). When two si*Lamp2a* oligos were used to knockdown Lamp2a, Ataxin-10 also accumulated (Fig. 5, *J* and *K*). These results indicate that Ataxin-10 is a client protein of the CMA pathway.

As these results suggest that O-GlcNAcylation is essential for HSC70 to interact with CMA substrates, we explored whether it holds true to other known CMA clients, such as ULK1 (20) (Fig. S3). We found that the glycosylation-deficient HSC70 mutant attenuated association with ULK1 significantly, consistent with our hypothesis. Moreover, we used other cell lines (HeLa cells) (Fig. S4) to examine whether it also applies in more physiologically relevant settings. The results showed that HSC70-T430A also downregulated interaction with PKM2 and Ataxin-10 in HeLa cell lines. In sum, T430 O-GlcNAcylation is indispensable for HSC70 to bind with CMA partners.

### Identification of a KFERQ-like motif of Ataxin-10

**Figure 6:**
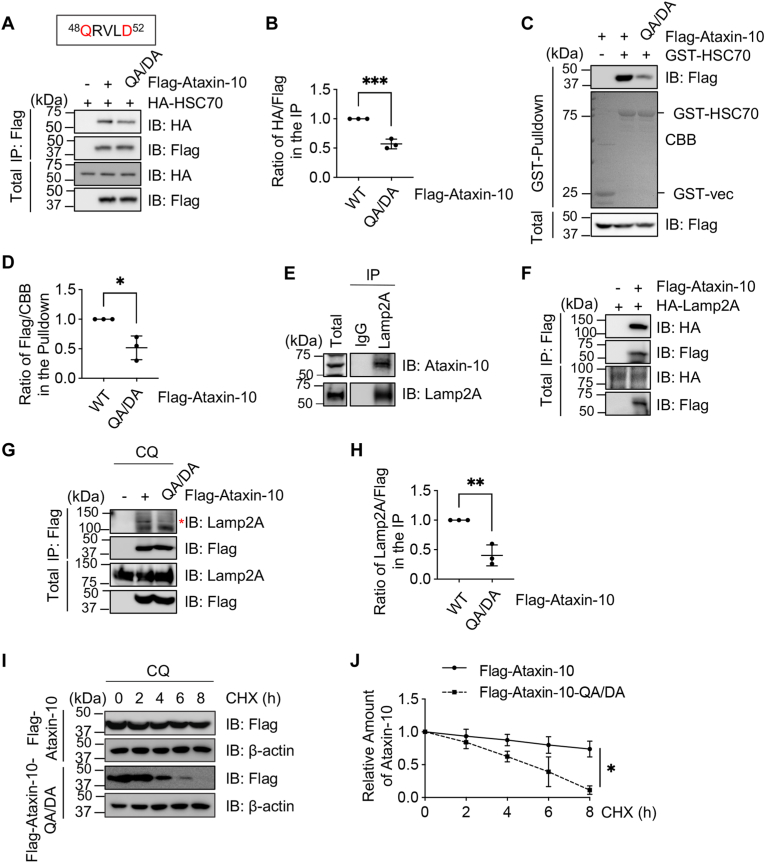
**Identification of a potential KFERQ motif of Ataxin-10.***A*, HEK-293T cells were transfected with Flag-Ataxin-10, -Q48A/D52A (QA/DA) and HA-HSC70, and then the lysates were immunoprecipitated and immunoblotted with the antibodies indicated. *B*, the quantitation of (*A*). *C*, cells were transfected with Flag-Ataxin-10 and -QA/DA plasmids. Recombinant GST-HSC70 proteins were incubated with the cellular lysates, and GST pull-down experiments were carried out. *D*, the quantitation of (*C*). *E*, co-IP between endogenous Lamp2A and Ataxin-10. *F*, cells were transfected with HA-Lamp2A and Flag-Ataxin-10 plasmids. *G*, cells were transfected with Flag-Ataxin-10 and -QA/DA plasmids and treated with 10 μM chloroquine (CQ) for 3 h. *Red asterisk* indicates the band of interest. *H*, the quantitation of (*G*). *I*, CHX pulse-chase assays. Cells were transfected with Flag-Ataxin-10 or -QA/DA plasmids, then treated with CQ and CHX for different durations. *J*, the quantitation of (*I*). Quantitation in (*J*) was carried out with two-way ANOVA. Quantitation in (*B*), (*D*), and (*H*) were carried out with Student’s *t* test. ∗*p* < 0.05; ∗∗*p* < 0.01; ∗∗∗*p* < 0.001. CHX, cycloheximide; co-IP, co-immunoprecipitation; HSC70, heat shock cognate protein 70; IP, immunoprecipitation.

We sought to identify the potential KFERQ-like motif of Ataxin-10, with one such motif predicted to be ^48^QRVLD^52^ (KFERQ finder V0.8, https://rshine.einsteinmed.edu). We first generated *ATXN10*-Q48A/D52A (*ATXN10*-QA/DA) mutants, and then observed that the mutants bound less HSC70 in cells (Fig. 6, *A* and *B*). We then used the GST-pull-down assay. Consistently, recombinant GST-HSC70 proteins pulled-down less Flag-Ataxin-10-QA/DA proteins (Fig. 6, *C* and *D*), suggesting that ^48^QRVLD^52^ is a major KFERQ for the association between HSC70 and Ataxin-10.

We further tested the affinity between Ataxin-10 and Lamp2A, the CMA receptor. As illustrated in Figure 6*E*, Lamp2A interacts with endogenous Ataxin-10. On overexpression, HA-Lamp2A interacts with Flag-Ataxin-10 (Fig. 6*F*). However, the QA/DA mutant showed reduced affinity with Lamp2A upon lysosome inhibition by chloroquine (CQ) (Fig. 6, *G* and *H*), suggesting that ^48^QRVLD^52^ increases the interaction of Ataxin-10 with HSC70 and Lamp2A.

We then sought to examine whether this KFERQ-like motif targets Ataxin-10 to the lysosome for degradation. As shown in Figure 6, *I* and *J*, cells were incubated with CQ and pretreated CHX as described before (20, 21, 22). Although this treatment induced a marked decrease in Flag-Ataxin-10-QA/DA protein stability, Flag-Ataxin-10-WT proteins remained stable, suggesting that Ataxin-10-WT is mainly degraded *via* CMA, while Ataxin-10-QA/DA is degraded *via* the ubiquitin-proteasome pathway. These data indicate that Ataxin-10 can be degraded by CMA through the ^48^QRVLD^52^ motif.

## Discussion

**Figure 7:**
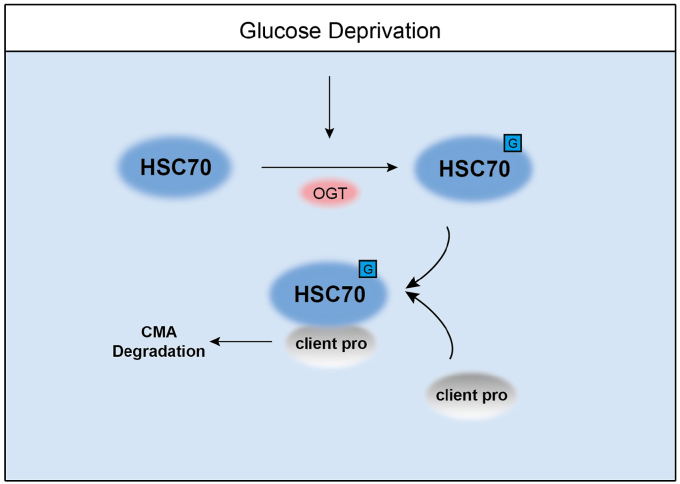
**Working model.** Upon glucose deprivation, the interaction between HSC70 and OGT increases, thus promoting HSC70 O-GlcNAcylation at T430, which in turn upregulates the CMA pathway for client protein degradation. CMA, chaperone-mediated autophagy; HSC70, heat shock cognate protein 70; O-GlcNAc, O-linked β-N-acetylglucosamine; OGT, O-GlcNAc transferase.

CMA is a cellular response to starvation to provide nutrients for energy regeneration and protein synthesis, but it is largely unknown what is the CMA activation signal (13). Here, we tried to address what activates CMA upon sugar depletion. As glucose deprivation is known to upregulate O-GlcNAcylation, we sought to uncover the link between O-GlcNAcylation and CMA. We then identified that low glucose induces HSC70 O-GlcNAcylation at T430, which is fundamental for CMA substrate association (Fig. 7). Using label-free quantitative MS, we further found a new CMA client, Ataxin-10, which is a protein involved in a neurodegenerative disease. Our results suggest that HSC70 O-GlcNAcylation contributes partly to CMA activation. Thus, our work links CMA activation and O-GlcNAcylation, which is a signal in response to nutrient status.

Dysregulation of proteolysis is widely accepted to be involved in neurodegenerative disease pathogenesis, and CMA in particular, is also implicated in Parkinson’s disease (PD) and Hungtington’s disease (HD) (34). PD is characterized by the aggregation of α-synuclein. The connection between CMA and PD has long been appreciated: CMA inhibition elevates ubiquitinated α-synuclein (35), while CMA activation counteracts the toxicity of α-synuclein accumulation (36). Other hereditary forms of PD have also been linked to protein mutations that inactivate CMA (37). HD is caused by polyglutamine expansion in the N terminus of Huntington (Htt), which caused its aggregation. Htt is a substrate protein of CMA (38). As mutant Hunting disrupts macroautophagy, CMA is elevated as a compensatory mechanism (39). Consequently, both HSC70 and Lamp2a are upregulated (39). With the ageing process, the compensatory CMA upregulation is diminished, which correlates with an exacerbation of HD (39).

Our results are interesting in two aspects. First, it is known that O-GlcNAcylation declines as humans age. It is conceivable that HSC70 O-GlcNAcylation would reduce as we grow older, which may contribute partially to the diminished compensatory CMA upregulation in HD patients. Second, we identified a new CMA substrate, Ataxin-10, which is a protein involved in SCA10, a neurodegenerative disease characterized by progressive pancerebellar ataxia and epilepsy (40, 41). The disease-causing expanded introns are shown to form mini-dumbbell structures and escape from the proofreading function of DNA polymerase (42). They also aggregate, colocalize with and suppress hnRNP K, a major RNA binding protein (43). Subsequently, PKC δ translocates to the mitochondrial and activates Caspase-3, leading to neuronal death (43). But the regulation of the Ataxin-10 protein remains unresolved. Previously, our group showed that Ataxin-10 fine-tunes cytokinesis (31, 32) and partially localizes in the Golgi (33). Using *atxn10* mutant mouse models, Ataxin-10 is also found to be a prerequisite for embryonic heart development and epithelial cell maintenance in the kidney and pancreas (44). Here, we demonstrate that Ataxin-10 is a CMA substrate, it would be interesting to explore the effects of Ataxin-10 proteolysis in SCA10 patient cells.

It is paradoxical that O-GlcNAcylation attenuates HSC70 stability while elevating its selectively. It is conceivable that the nonspecific binding between HSC70 and nonsubstrate proteins are weakened and this portion of HSC70 is degraded. But how this specificity is achieved warrants further investigation. In sum, we found that starvation-induced HSC70 O-GlcNAcylation activates CMA, which may have implications for neurologic disease pathogenesis and progression.

## Experimental procedures

### Cell culture and antibodies

HEK-293T cells were purchased from American Type Culture Collection. The cell lines were validated using short tandem repeat profiling and were free from *mycoplasma* contamination for all experiments. Antibodies were as follows: anti-Flag antibody (Sigma-Aldrich, #F1084), anti-HA antibody (ABclonal, #AE105), anti-IgG Rabbit antibody (Sigma-Aldrich, #R2665), anti-RL2 antibody (Abcam, #ab2739), anti-Myc antibody (PTM BIO, #PTM-5390), anti-HSC70 (Proteintech, #10654-1-AP), anti-β-actin antibody (Sigma-Aldrich, #A5441), anti-Ataxin-10 antibody (Bethyl, A301–054A), anti-Lamp2A antibody (Abcam, #ab18528). si*HSC70* UTR: GAACAAGA GAGCTGTAAGA, GTGCCATGACAAAGGATAA (22). si*Lamp2a*: CTCCTCTATTTGCTAAGTA, GCTTCAACTCCAATAAGAT.

### Immunoprecipitation and immunoblotting

Immunoprecipitation and immunoblotting (IB) experiments were performed as described before (32). The following primary antibodies were used for IB: anti-Flag (1:4000), anti-HA (1:4000), anti-RL2 antibody (1:1000), anti-Myc antibody (1:2000), anti-HSC70 (1:1000), anti-β-actin (1:4000), anti-Ataxin-10 (1:2000), anti-Lamp2A (1:1000). LAS-4000 was employed to detect signals and quantitated by the Multi Gauge software (Fujifilm).

### Glucose deprivation

Glucose deprivation experiment was performed as described before (8). Briefly, cells were washed twice with 1×PBS, and then incubated in the glucose-free Dulbecco’s modified Eagle’s medium (Gibco, #11966025) supplemented with 10% (v/v) fetal bovine serum and penicillin/streptomycin for 24 h.

### Chemicals

CQ (MCE, HY-17589A) was used at 10 μM for 3 h; CHX (Sigma-Aldrich, C7698-5G) was used at 150 mg/ml for different times; Thiamet-G (TMG) (OGA inhibitor) (MedChemExpress, #HY-12588) was used at 5 mM for 24 h; acetyl-5S-GlcNAc (5S) (OGT inhibitor) was used at 100 mM (prepared at 50 mM in dimethyl sulfoxide) for 24 h.

### Biorthogonal chemistry assays

Biorthogonal chemistry experiments were performed as described before (25). Cells were treated with 200 μmol/L Ac_3_6AzGlcNAc for 24 h. Collected cells were lysed with 150 mM lysis buffer (150 mM NaCl, 1 M Tris (pH 7.5), 0.5 M EDTA, and 10% NP-40) containing a protease inhibitor cocktail (Roche) for 1 h at 4 °C. Next, cell lysates were cleared using centrifugation (4 °C; 12,000 rpm; 10 min). The supernatant reacted with 50 μmol/L DBCO-PEG_4_-Biotin from TargetMol Chemicals Inc, 8 mmol/L urea, 10 mmol/L Hepes (pH 7.9) and Halt Protease & Phosphatase Inhibitor Cocktail (100 × ) from Thermo Fisher Scientific, then the pull-down complex isolated by streptavidin-coupled beads was subjected to Western blotting analysis.

### Label-free quantitative mass spectrometry

#### Sample preparation

The acetone-precipitated protein pellets were fully dissolved in 8 M urea, incubated in 10 mM DTT in 50 mM ammonium bicarbonate at 37 °C for 45 min, then incubated in 10 mM iodoacetamide in 50 mM ammonium bicarbonate at ambient temperature for 1 h in the dark. The solution was then diluted to a urea concentration of 2 M using 50 mM ammonium bicarbonate, followed by trypsin digestion with an enzyme:protein ratio of 1:40 at 37 °C overnight. Formic acid was added to the solution to a final concentration of 0.1% to quench the digestion. All samples were vacuum-centrifuged to dryness, and resuspended in 0.1% formic acid in water prior to liquid chromatography-tandem mass spectrometry (LC-MS/MS) analysis.

#### Liquid chromatography-tandem mass spectrometry parameters

Peptides were separated using a loading column (100 μm × 2 cm) and a C18 separating capillary column (75 μm × 15 cm) packed in-house with Luna 3 μm C18(2) bulk packing material (Phenomenex). The mobile phases (A: water with 0.1% formic acid and B: 80% acetonitrile with 0.1% formic acid) were driven and controlled by a Dionex Ultimate 3000 RPLC nano system (Thermo Fisher Scientific). The LC gradient was held at 2% for the first 8 min of the analysis, followed by an increase from 2 to 10% B from 8 to 9 min, an increase from 10 to 44% B from 9 to 63 min, and an increase from 44 to 99% B from 63 to 68 min.

For the samples analyzed by Orbitrap Fusion LUMOS Tribrid Mass Spectrometer, the precursors were ionized using an EASY-Spray ionization source (Thermo Fisher Scientific) source held at +2.0 kV compared to ground, and the inlet capillary temperature was held at 320 °C. Survey scans of peptide precursors were collected in the Orbitrap from 350 to 1600 Th with an AGC target of 400,000, a maximum injection time of 50 ms, RF lens at 30%, and a resolution of 60,000 at 200 m/z. Monoisotopic precursor selection was enabled for peptide isotopic distributions, precursors of z = 2 to 7 were selected for data-dependent MS/MS scans for 3 s of cycle time, and dynamic exclusion was set to 15 s with a ±10 ppm window set around the precursor monoisotope.

In higher-energy collisional dissociation scans, an automated scan range determination was enabled. An isolation window of 1.6 Th was used to select precursor ions with the quadrupole. Product ions were collected in the Orbitrap with the first mass of 110 Th, an AGC target of 50,000, a maximum injection time of 30 ms, higher-energy collisional dissociation collision energy at 30%, and a resolution of 15,000.

## Data analysis

Data processing was carried out using Thermo Proteome Discoverer 2.4 using a SwissProt *Homo sapiens* database (TaxID=9606 and subtaxonomy, 42,253 protein sequences). Carbamidomethyl (Cys) were chosen as static modification, and oxidation (Met) was chosen as variable modification. Mass tolerance was 10 ppm for precursor ions and 0.02 Da for fragment ions. Maximum missed cleavages were set as 2. Peptide spectral matches were validated using the Percolator algorithm, based on q values at a 1% false discovery rate. For label-free quantitation, normalization mode was set to “total peptide amount”. The protein abundances were calculated by summing sample abundances of the corresponding peptides after normalization.

## Data availability

The mass spectrometry proteomics data have been deposited to the ProteomeXchange Consortium *via* the PRIDE (45) partner repository with the dataset identifier PXD070146 and 10.6019/PXD070146.

## Supporting information

This article contains supporting information.

## Conflict of interest

The authors declare that they have no conflicts of interest with the contents of this article.
