# Supplementary material for: Starvation-induced HSC70 O-GlcNAcylation activates chaperone-mediated autophagy
Source: J Biol Chem. 2026 May 15;302(7):113165. doi: 10.1016/j.jbc.2026.113165 (PMC13273674; doi:10.1016/j.jbc.2026.113165)
Supplement: Supplementary Material [file mmc2.pdf]

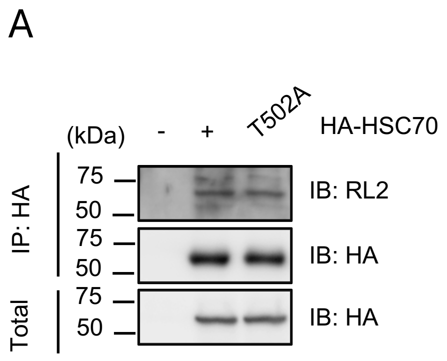

Figure. S1 Residue T502 is not a critical site for the O-GlcNAcylation of HSC70.
A, HEK-293T cells were transfected with HA-HSC70-WT and HA-HCS70-T502A plasmids, and then the lysates were immunoprecipitated and immunoblotted with the antibodies indicated.

Supplementary Fig 1

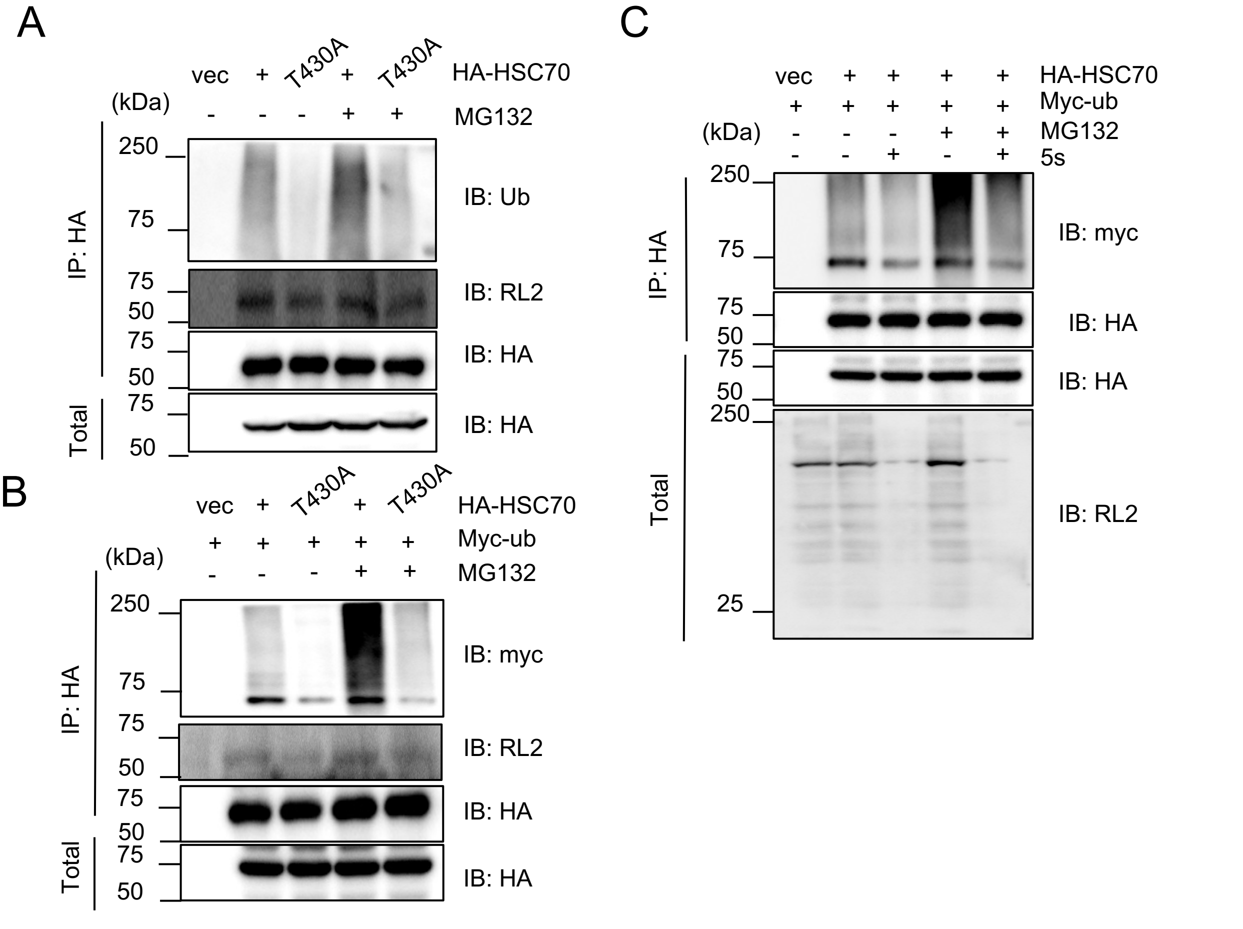

Figure. S2 HSC70 O-GlcNAcylation promotes ubiquitination.

A, HEK-293T cells were transfected with HA-HSC70-WT, -T430A plasmids, then treated with 10 µmol/L MG123 or not, and then the lysates were immunoprecipitated and immunoblotted with the antibodies indicated.

B, cells were transfected with HA-HSC70-WT ,-T430A and Myc-Ub plasmids, then treated with 10 µmol/L MG132 or not treated.

C, cells were transfected with HA-HSC70-WT and Myc-Ub, then treated with 10 µmol/L MG132 , 50 µmol/L acetyl-5S-GlcNAc (5S, OGT inhibitor) or not treated.

Supplementary Fig 2

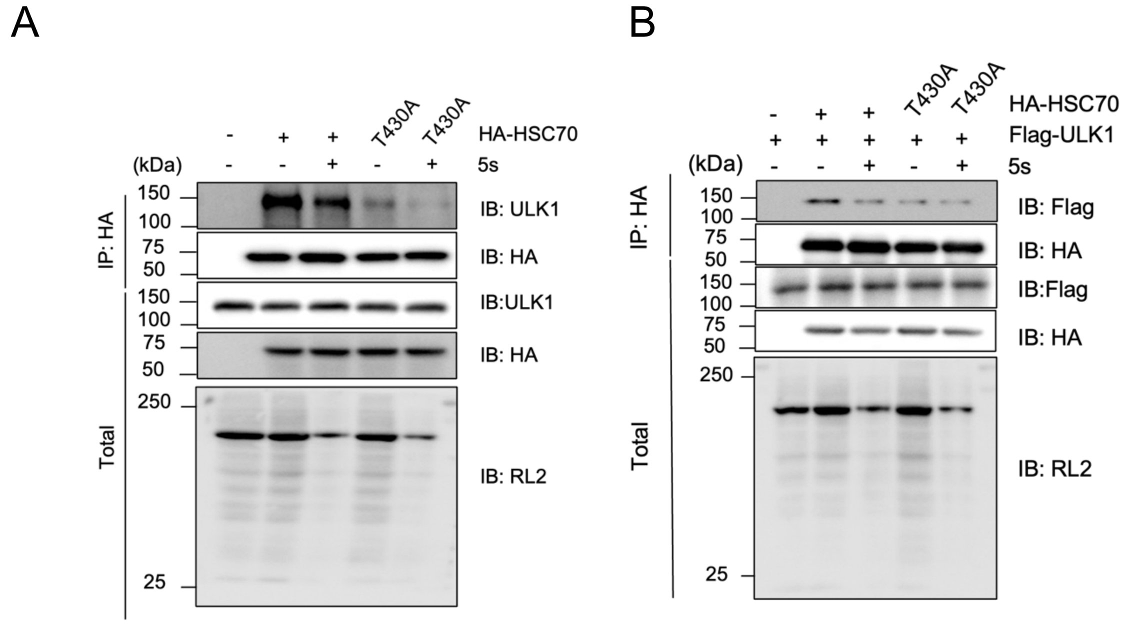

Figure. S3 HSC70 O-GlcNAcylation promotes interaction ULK1.
A, HEK-293T cells were transfected with HA-HSC70-WT and HA-HSC70-T430A plasmids, then treated with 50 μmol/L acetyl-5S-GlcNAc (5S, OGT inhibitor) or not, and then the lysates were immunoprecipitated and immunoblotted with the antibodies indicated.
B, cells were transfected with HA-HSC70-WT ,-T430A and Flag-Ataxin-10 plasmids, then treated with 5s or not.

Supplementary Fig 3

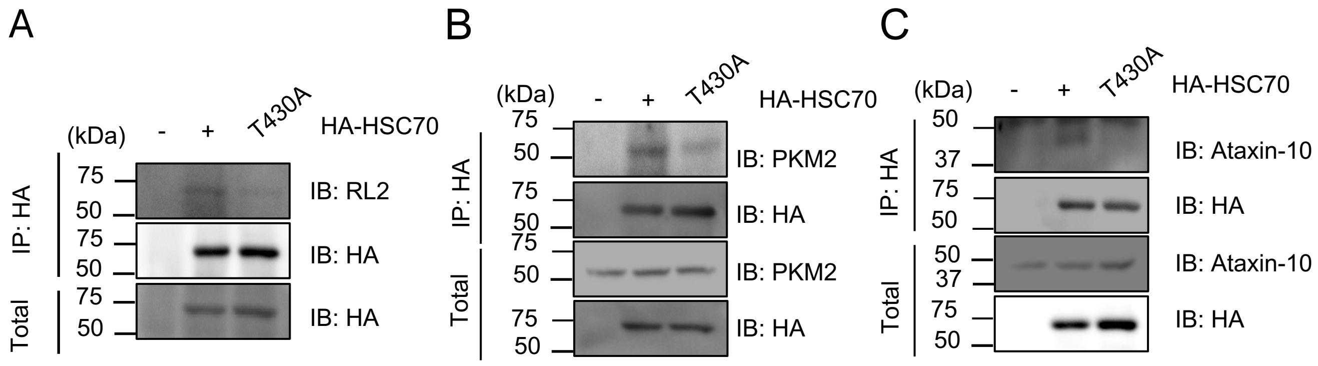

Figure. S4 O-GlcNAcylation of HSC70 at Thr430 promotes its interaction with PKM2 and Ataxin-10 in HeLa cells.

A, B and C, HeLa cells were transfected with HA-HSC70-WT and HA-HCS70-T430A plasmids, and then the lysates were immunoprecipitated and immunoblotted with the antibodies indicated .

Supplementary Fig 4
